# Supplementary material for: Mapping brain-wide excitatory projectome of primate prefrontal cortex at submicron resolution and comparison with diffusion tractography
Source: eLife. 2022 May 20;11:e72534. doi: 10.7554/eLife.72534 (PMC9122499; doi:10.7554/eLife.72534)
Supplement: Transparent reporting form [file elife-72534-transrepform1.docx]

***eLife’s* transparent reporting form**

We encourage authors to provide detailed information *within their submission* to facilitate the interpretation and replication of experiments. Authors can upload supporting documentation to indicate the use of appropriate reporting guidelines for health-related research (see [EQUATOR Network](http://www.equator-network.org/%20)), life science research (see the [BioSharing Information Resource](https://biosharing.org/)), or the [ARRIVE guidelines](http://www.plosbiology.org/article/info:doi/10.1371/journal.pbio.1000412) for reporting work involving animal research. Where applicable, authors should refer to any relevant reporting standards documents in this form.

If you have any questions, please consult our Journal Policies and/or contact us: [editorial@elifesciences.org](mailto:editorial@elifesciences.org).

**Sample-size estimation**

- You should state whether an appropriate sample size was computed when the study was being designed
- You should state the statistical method of sample size computation and any required assumptions
- If no explicit power analysis was used, you should describe how you decided what sample (replicate) size (number) to use

Please outline where this information can be found within the submission (e.g., sections or figure legends), or explain why this information doesn’t apply to your submission:

For virus testing, to ensure our observations are reproducible, we included at least 3 injection sites for each viral vector.

For the parts of vlPFC projectome tracing and comparison between dMRI and STP, we aimed to reconstruct the mesoscale excitatory projectome issued from the ventrolateral prefrontal cortex (vlPFC) and introduce an analysis pipeline for integration of connectomic data from microscopy to MRI in macaque. Analogous to most tracer-injection studies, here for the descriptive and quantitative results presented in this work statistical testing is not applicable. A sample size estimation also not required.

For more details please see Table 1.

**Replicates**

- You should report how often each experiment was performed
- You should include a definition of biological versus technical replication
- The data obtained should be provided and sufficient information should be provided to indicate the number of independent biological and/or technical replicates
- If you encountered any outliers, you should describe how these were handled
- Criteria for exclusion/inclusion of data should be clearly stated
- High-throughput sequence data should be uploaded before submission, with a private link for reviewers provided (these are available from both GEO and ArrayExpress)

Please outline where this information can be found within the submission (e.g., sections or figure legends), or explain why this information doesn’t apply to your submission:

Microscopic imaging and dMRI imaging were performed once per animal. Details of virus injection experiment, including expression time for each viral vector, can be found in Table 1.

**Statistical reporting**

- Statistical analysis methods should be described and justified
- Raw data should be presented in figures whenever informative to do so (typically when N per group is less than 10)
- For each experiment, you should identify the statistical tests used, exact values of N, definitions of center, methods of multiple test correction, and dispersion and precision measures (e.g., mean, median, SD, SEM, confidence intervals; and, for the major substantive results, a measure of effect size (e.g., Pearson's r, Cohen's d)
- Report exact p-values wherever possible alongside the summary statistics and 95% confidence intervals. These should be reported for all key questions and not only when the p-value is less than 0.05.

Please outline where this information can be found within the submission (e.g., sections or figure legends), or explain why this information doesn’t apply to your submission:

Pearson Correlation Coefficients were used for statistically assessing the spatial overlap between each pair of axonal density map and probabistic map. Pearson coefficients ranging from + 1 (perfect positive correlation) to 0 (no linear correlation) to− 1 (perfect anti-correlation). The statistical analysis was performed pixel by pixel, and the P values were calculated to evaluate the significance of the correlation. Those statistical tests were reported in Results, subsection “Comparison of vlPFC axonal projections by dMRI and STP”, and in Figure 6.

(For large datasets, or papers with a very large number of statistical tests, you may upload a single table file with tests, Ns, etc., with reference to sections in the manuscript.)

**Group allocation**

- Indicate how samples were allocated into experimental groups (in the case of clinical studies, please specify allocation to treatment method); if randomization was used, please also state if restricted randomization was applied
- Indicate if masking was used during group allocation, data collection and/or data analysis

Please outline where this information can be found within the submission (e.g., sections or figure legends), or explain why this information doesn’t apply to your submission:

For virus testing in macaque brain, experimental groups were defined by genotype of viral constructs.

For vlPFC projectome tracing, group allocation is not applicable.

For comparison between dMRI and STP, experimental groups were defined by image modalities.

**Additional data files (“source data”)**

- We encourage you to upload relevant additional data files, such as numerical data that are represented as a graph in a figure, or as a summary table
- Where provided, these should be in the most useful format, and they can be uploaded as “Source data” files linked to a main figure or table
- Include model definition files including the full list of parameters used
- Include code used for data analysis (e.g., R, MatLab)
- Avoid stating that data files are “available upon request”

Please indicate the figures or tables for which source data files have been provided:

The source data for Figure 3 were provided as a Supplementary File.

There is no publicly accessible resource for hosting such big connectome data. Therefore we host it ourselves on an institutional FTP server which can be accessed via username and password (available upon request). We commit to keeping it available for at least 5 years, and provide alternative procedures where users can copy any or all of it to their own computer if needed.
